# Peruvian healthcare system readiness for dementia

**DOI:** 10.1002/alz70860_097543

**Published:** 2025-12-23

**Authors:** Francisco Tateishi Serruto, Maria Lazo Porras, Daniela Rossini Vilchez, Antonio Bernabe, Christopher R. Butler, María Sofía Cuba, Graham Moore, Francisco Diez Canseco, Filipa Landeiro, Maria Kathia Cardenas, Carlos Vera Tudela, Jemma Hawkins

**Affiliations:** ^1^ Cronicas, Universidad Peruana Cayetano Heredia, LIma, Lima, Peru; ^2^ CRONICAS, Universidad Peruana Cayetano Heredia, Lima, Lima, Peru; ^3^ CRONICAS Centro de excelencia en enfermedades crónicas ‐ Universidad Peruana Cayetano Heredia, Lima, Lima, Peru; ^4^ Department of Brain Sciences, Imperial College London, UK, London, London, United Kingdom; ^5^ Universidad Peruana Cayetano Heredia, Lima, Lima, Peru; ^6^ Cardiff University, Cardiff, Wales, United Kingdom; ^7^ University of Oxford, Oxford, Oxfordshire, United Kingdom; ^8^ CRONICAS, Universidad Peruana Cayetano Heredia, Lima, Peru; ^9^ Cardiff University, Cardiff, United Kingdom

## Abstract

**Background:**

The prevalence of dementia in low and middle income countries is rising dramatically, aggravated by low socioeconomic status and poor educational levels, as seen in Peru, where 41.6% of older adults in rural areas are illiterate. This limited literacy can hinder early recognition and management of dementia. The IMPACT Salud project, aligned with WHO's 2017 Global Action Plan (GAP) for Dementia and the Peruvian Ministry of Health's efforts to prioritise dementia as a public health issue. This study is Peru's first initiative to assess the health system's capacity to diagnose, treat, and support people with dementia (PWD) and their carers.

**Method:**

Between July 2023 and August 2025, we conducted a health system assessment through a qualitative study involving stakeholder interviews and documentation review, using a methodology appropriate for low‐ and middle‐income settings. Twelve stakeholder types across three health system levels participated in four Peruvian regions. These included PWD, carers, health professionals, regional managers, and policymakers. Our analysis used the High‐Quality Health System Framework, which evaluates three domains: foundations, process of care, and quality impacts.

**Result:**

We interviewed 160 participants, including PWD, carers, health providers, and policymakers. Preliminary findings indicate: Foundations: There is limited political support for dementia care, no institutionalized standard protocols, few medications in public pharmacies, no training programs, and no financial support. Only one hospital in one of the four regions has specific PWD care protocols. Process of Care: A referral system is in place but overloaded, delaying timely access to specialized consultations and imaging. PWD and carers judge care quality based on the empathy shown by health workers. In Lima, PWD have better specialist access and receive faster diagnoses. Quality Impacts: The trust PWD and carers place in the health system depends on care level and staff empathy. They perceive health improvements primarily in reduced symptoms and easier home care, rather than changes in cognitive decline.

**Conclusion:**

The data analysed so far suggest that the Peruvian health system is not currently prepared to provide quality care that meets the needs of PWD and their carers. To follow the GAP recommendations is essential to properly implement Peru's dementia law, ensure high‐quality services and consider regional differences.

